# Assessment of replicate bias in 454 pyrosequencing and a multi-purpose read-filtering tool

**DOI:** 10.1186/1756-0500-4-149

**Published:** 2011-05-26

**Authors:** Mariette Jérôme, Céline Noirot, Christophe Klopp

**Affiliations:** 1 Plate-forme bio-informatique Genotoul, INRA, Biométrie et Intelligence Artificielle/Génétique Cellulaire, BP 52627, 31326 Castanet-Tolosan Cedex, France

## Abstract

**Background:**

Roche 454 pyrosequencing platform is often considered the most versatile of the Next Generation Sequencing technology platforms, permitting the sequencing of large genomes, the analysis of variations or the study of transcriptomes. A recent reported bias leads to the production of multiple reads for a unique DNA fragment in a random manner within a run. This bias has a direct impact on the quality of the measurement of the representation of the fragments using the reads. Other cleaning steps are usually performed on the reads before assembly or alignment.

**Findings:**

PyroCleaner is a software module intended to clean 454 pyrosequencing reads in order to ease the assembly process. This program is a free software and is distributed under the terms of the GNU General Public License as published by the Free Software Foundation. It implements several filters using criteria such as read duplication, length, complexity, base-pair quality and number of undetermined bases. It also permits to clean flowgram files (.sff) of paired-end sequences generating on one hand validated paired-ends file and the other hand single read file.

**Conclusions:**

Read cleaning has always been an important step in sequence analysis. The pyrocleaner python module is a Swiss knife dedicated to 454 reads cleaning. It includes commonly used filters as well as specialised ones such as duplicated read removal and paired-end read verification.

## Findings

NGS platforms are now well implanted in sequencing centers and some laboratories. The hundred fold decrease of sequencing costs widens the scientific population accessing this type of data. The reads produced by NGS platforms are prone to errors, some of which are random and others specific to a given platform often called biases. Cleaning bad quality or biased reads is often the first step before any other analysis. Several pieces of software exist but they are specialised in some filters. Tools such as SeqClean [2], lucy [3] or Figaro [4] gather common but useful filters based on different criteria such as length, quality, polya tails, complexity, vector presence, contamination. Another like cd-hit-454 [5] specifically discards artificial duplicated reads [6] observed in pyrosequencing runs. The purpose of pyrocleaner is to offer a tool as complete as possible to clean and assess 454 reads.

### Implementation

PyroCleaner aims to clean duplicated reads generated by the Roche 454 platform in a controlled way as close as possible to a random read selection process. Replicate seeking (*--clean-duplicated-reads *option) relies on an alignment of all sequences against themselves, using megablast [7]. The result is used to build a graph connecting similar reads. In this graph, each read is represented by a vertex and edges represent the similarities between reads. Edges are created only if the similarity between read starts at the first nucleic position of both reads, has a score higher than 100 and if both reads have the same strand. Due to the homopolymer bias and sequencing errors, duplicated reads can offer slight differences:insertions, deletions or substitutions and they can stop at different positions. Two options are available regarding the aggressiveness of the duplicated read removal step. Using the aggressive option (--aggressive), two reads are connected disregarding the read length. Without the aggressive option, the algorithm will connect reads only if their length differences are lower than a given threshold value (default is 70 bases). Connected components are then extracted using the igraph library [8]. Each component represents a duplication cluster of which only the longest read is kept in the result file. The underlying idea is that artificial duplicates will be much more alike than reads coming from two different fragments starting at the same position.

**Figure 1:**
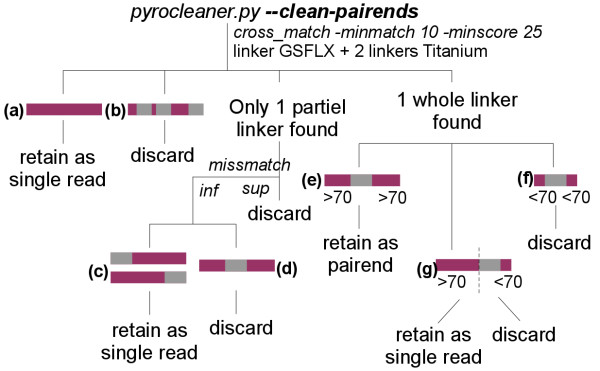
**Paired-end cleaning strategy**. Reads having no linker (a) are retained as single reads. If multiple linkers are present (b) in the same read, the read is discarded. In cases where the linker is partially found, meaning that the number of mismatches is lower than a threshold, only reads where the linker is located at the beginning or at the end (c) are saved as single reads, others (d) are deleted. Reads where the entire linker is present and not to closely located to one end (e) are saved as paired-end reads. In other cases, sequences are saved as single reads only if the linker is located far enough from one end (g), while others (f) are deleted.

The module also provides an option to filter paired-end reads:*--clean-pairends*. A 454 paired-end read should be composed of the sequence of one end of the DNA fragment, a linker sequence and the sequence of the other end of the DNA fragment. Unfortunately in some cases the linker is missing. In other cases the linker is too close to the end of the read and therefore the mate-pair cannot be used to bridge contigs in an assembly process. Cleaning paired-end reads relies on seeking this linker. The Roche platform uses three different linkers depending on the chemistry, one for GSFLX and two others for Titanium. Using the option generates a local similarity search which is performed between input sequences and 454 linkers using cross_match [9]. It leads to the generation of two output files using the strategy presented in Figure 1. The first file will contain all good quality paired-end reads. The second one gathers all reads in which the linker was missing, the linker location too close to one end or the linker sequence quality too low. In the last two cases the reads are clipped in order to keep the longest subsequence without linker. Thus, all reads from the second file can be used as single reads in the assembly.

More basic but useful cleaning options are also provided. Discarding reads using their length can be done by setting min/max values (*--clean-length-win *option) or by using the standard deviation (*--clean-length-std *option) computed on all input reads. Reads can be filtered based on their complexity, which is computed using the compressed string length (library zip) on the complete sequence (*--clean-complexity-full *option) or on several sub-sequences generated using a sliding window approach (*--clean-complexity-win *option). In the latter last case, the read is flagged as complex if at least one sub-sequence complexity is higher than the given threshold. Reads can also be discarded if none of its base pairs has a quality value above the given threshold (--clean-quality-full option) or if the rate of undetermined bases is higher than a specified value (--clean-ns option). Pyrocleaner produces several output files. The result files containing the reads can be written into several formats such as sff, fastq or fasta. Exporting reads into sff format is convenient as it is now widely used by assemblers. However, exporting reads in this format depends on the *sfffile *script provided by Roche:if it is missing, the output will be written in the fastq format. The log file gives precise information about the cleaned reads and the reason of cleaning. The last lines of the generated log file contain cleaning summary and duplication profile figures.

## Results and Discussion

**Figure 2:**
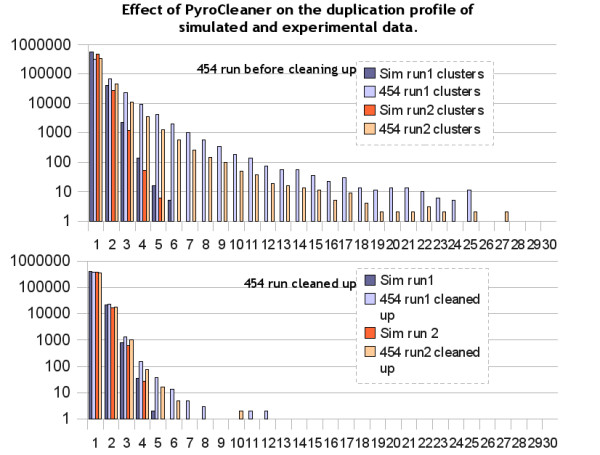
**Duplication profile before pyrocleaning and after**. Simulated dataset were produced using the *E coli *K12 genome. Sequences of 500 bp were picked randomly along the genome using both strands. The number of simulated sequences (Sim run1/Sim run2) equals the number of sequences produced in the experimental runs (454 run1/454 run2).

In order to analyse the PyroCleaner efficiency on duplication cleaning, we used a technical validation run produced in collaboration with Roche (data are available on the ecoli demonstration runs of the ng6 web site http://ng6.toulouse.inra.fr/). It was a whole genome sequencing of *E coli *K12 Titanium run where one half plate was prepared by a PlaGe sequencing platform [9] technician (Run1, see Figure 2) and the second half plate by a Roche technician, both using the same protocol (Run2, see Figure 2). The aim was to compare the duplication profile between real and simulated data. The simulated reads were picked randomly from both strands in the *E coli *K12 genome. Two simulations were performed. Both simulations have demonstrated a replication rate close to 8%, which is far from the 31% and 18% obtained respectively for the first and seconds runs.

For both 454 and simulated datasets the structure of duplication was produced using PyroCleaner log files. The probability of appearance of large clusters of duplicated reads is much higher with the 454 platform than with random selection. Figure 2a shows the distribution of duplicated read clusters with cluster size going from 2 to 30. With simulated data the largest cluster contains only five reads whereas clusters with as many as 27 reads are present in the experimental data. Figure 2b presents the structure of duplication calculated on the experimental runs once cleaned by PyroCleaner. It clearly reduces the number of large clusters and produces a read set having a structure which is close to random selection.

It took 123 minutes to process 671 856 *E coli *sequences on a quad-core machine with 32Gb of memory. 28 624 sequences were discarded because of their length (4.3%), 65 because of the number of undetermined bases, 21 807 because of their low complexity (3.2%), 663 because of their poor quality (0.1%) and 156 222 because they have been flagged as duplicate (23.3%).

The multiple read bias impact is closely linked to the type of analysis performed. As long as the fragmentation protocol produces a random selection of fragment start positions, the reads can easily be cleaned using the PyroCleaner. In case of non-random selection of sequence end positions like AFLP, 3' mRNA tags, and reduced representation libraries there is no solution to distinguish between artificially duplicated reads and sequences coming from multiple fragments starting at the same position.

## Competing interests

The authors declare that they have no competing interests.

## Authors' contributions

JM implemented, designed the tools and wrote the manuscript. CN participated in the implementation and the design. CK participated in its coordination and helped to draft the manuscript. All authors read and approved the final manuscript.
